# Dental caries and weight among children in Nuuk, Greenland, at school entry

**DOI:** 10.1080/22423982.2017.1311535

**Published:** 2017-05-24

**Authors:** Signe Sloth Madsen, Vicky Jenny Rebecka Wetterstrand, Michael Lynge Pedersen

**Affiliations:** ^a^ Queen Ingrid Healthcare Centre, Nuuk, Greenland; ^b^ Queen Ingrid Hospital, Nuuk, Greenland; ^c^ Greenland Center for Health Research, Institute of Nursing and Health Sciences, University of Greenland, Nuuk, Greenland

**Keywords:** Dental caries, dft, overweight, weight class, children, Greenland, Inuit, health care

## Abstract

**Objective**: To explore the possible association between weight class and prevalence of caries among children born 2005-2007, living in Nuuk, Greenland, at time of school entry.

**Design**: A cross-sectional register study based on data from electronic medical records(EMR) and oral health data from public health and dental care facilities.

**Methods**: Data from routine examinations of children at time of primary school entry, including height and weight, were obtained from the EMRs. Dental charts recording oral health and caries were collected from public dental healthcare service. The prevalence of caries was calculated as the proportion of included children with dft score (decayed and/or filled non-permanent teeth) ≥1.

**Results**: 55%(373/681) had relevant data recorded in EMRs and dental charts, and could be included in the study. The prevalence of dental caries was 57.1%(213/373). The prevalence of caries increased with higher weight class,but no statistically significant trend was observed(p=0.063).

**Conclusions**: Increasing prevalence of caries with increasing weight class was observed in this study. A linear trend could not be confirmed statistically. The high prevalence of caries and overweight indicate the need for continued focus on preventative initiatives and monitoring. A combined strategy targeting both caries and overweight may be considered.

## Introduction

Overweight and obesity are serious health issues. They affect all organ systems and are correlated with psychosocial disturbances such as depression and low self-esteem [1–3]. The affected individuals suffer from reduced quality of life and the risk of a shorter lifespan [2,4]. According to the World Health Organization, obesity has more than doubled worldwide since 1980, and is no longer a public health challenge confined to industrialised or high-income countries [5]. The epidemiologic trend applies to children and adolescents as well as adults [1–3].

In Greenland, the number of obese and overweight children and adolescents has been rising significantly during the past four decades [6]. In 2010, more than 20% of all Greenlandic children at school entry were either overweight (15.8%) or obese (6.8%) [4].

Caries and poor oral health are additional common health problems in Greenland, and have been since the introduction of processed food and fermented carbohydrates [7,8]. The prevalence of caries among children and adolescents in Greenland has been significantly higher than in other Nordic countries [8,9]. The most recent effort to address this problem was initiated in 2008 when a national caries strategy was implemented, aiming to improve the oral health of Greenlandic children [9].

Both poor dental status and overweight are multifactorial conditions influencing the quality of life and development of children [10,11]. Both are complex, lifestyle-related health issues correlated with low socioeconomic status [12–14]. Important risk factors coincide in predisposing to both overweight and poor dental status, such as an inappropriate diet with high or frequent intake of sugar and soft drinks [14–17]. The preventative efforts needed in order to reduce the number of overweight and obese children and to improve oral health in the same age group might overlap and may demand a coordinated multidisciplinary strategy.

However, diverging correlations between overweight and caries have been reported internationally, and the possible association between weight for height and dental health among children in Greenland remains unexplored [13,14,16–20]. Thus, the aim of this study was to investigate the possible association between weight class and the prevalence of caries among Greenlandic children in Nuuk at school entry.

## Methods

This study was performed as a cross-sectional register study based on data from electronic medical records (EMRs) and dental charts from the dental service in Nuuk, Greenland.

### Setting

Nuuk is the capital of Greenland. With 16,500 inhabitants, it is also the largest city in the country, with all facilities expected from a modern metropole – including educational and health care centres. All children in Greenland receive a minimum of 10 years of primary school education. Before and during that time, they go through routine health examinations. They are examined by a midwife and a doctor at birth, and are offered regular examinations by a doctor when they turn 5 months, 12 months and 4 years of age. At 6 years of age, during enrolment in primary school, the children are examined by specialised nurses who, among other parameters, record the children’s height and weight. Data are recorded in the EMRs and used by the primary health care services in Nuuk.

Dental status is an important part of the overall health assessment, and as part of the national caries strategy in Greenland, children attend dental status check-ups at the ages of 8, 14 and 26 months and of 3, 4.5, 6, 9, 12 and 15 years [21]. Dentists in Greenland are educated and trained in Denmark. In collaboration with Greenlandic dental hygienists and dental assistants, they use dental charts with optical character recognition in order to register dental health at each oral examination. In doing this, the teams adhere to the internationally recognised dft/s and DMFT/S classification systems. By noting the number of decayed, missing and filled primary teeth (dft), a dft score can be calculated. For permanent teeth, the registration will be in capital letters. If surfaces are registered instead of teeth, the abbreviations will be respectively dmfs and DMFS  [22,23]. Caries and oral health conditions are registered manually on a dental chart for each child. The medical and dental examinations are offered by the public health care system in Greenland, approved by and funded through the government of Greenland. All families and households with children are encouraged to seize these opportunities for health care and prevention of disease, free of charge. To participate, as in any kind of public health care programme, a child must be accompanied by or have been given consent from at least one parent or the person in custody of the child.

### Study population

The study population was defined as all children with a permanent address in Nuuk, born in the years 2005–2007, who at school enrolment around at the age of 6 years attended routine health examinations with the registration of height and weight in their EMRs. Children with missing data on height or weight were not included in the study. In order to compare weight and dental status, dental charts on all included children were collected from the dental service in Nuuk. Children with missing dental charts were also excluded from the study.

### Variables

Weight was recorded in kilograms, and the children were weighed wearing light indoor clothing and no shoes. Height was measured in metres with the children wearing no shoes. Body mass index (BMI) was calculated by dividing the number of kilograms by height in metres squared (kg/m^2^). Children were categorised into thin, normal, overweight and obese based on the age- and gender-specific BMI limits recommend by the International Obesity Task Force [24–26].

A child was considered to have caries if a dft score of one or above was observed in the dental chart. Thus, the prevalence of caries was calculated as the proportion of included children with caries.

### Statistics

Statistical analysis was performed using SPSS version 23.0 (SPSS, Chicago, IL). Variables were described using medians and inter quartiles range (IQRs). Estimates were calculated with 95% confidence intervals. χ^2^ tests were used to compare frequencies and χ trend tests were used to analyse for linear trends among groups. The population in Nuuk as of 1 January 2010 was used as the background population. p-values below 0.05 were considered significant.

## Results

A total of 681 children (346 males and 335 females) born in the years 2005–2007 with a permanent address in Nuuk were identified. Of those, 509 had weight and height details recorded in their EMRs. Dental examination was performed in 373 of the 509 cases (185 males and 188 females). Thus, 55% (373/681) of all children born in 2005–2007 in Nuuk were included in the study. No difference in gender ratio was observed between included and not included children (p = 0.704). The basic variables for males and females are illustrated in Table 1. Males were slightly taller than females (p = 0.001). The proportion of males (84.9%) with normal weight was higher (p = 0.043) than females (76.6%). Otherwise, no other differences were observed between males and females. The prevalence of dental caries was 57.1% (213/373), with no gender difference observed (p = 0.731). The median dft score was 1.0 (IQR = 4.0) with a minimum of 0 and a maximum of 16. Table 2 illustrates the prevalence of caries for each weight category. The prevalence of caries increased with higher weight class, but the trend was not statistically significant (p = 0.063).

**Table 1.** Basic characteristics of the males and females included in the study.

	Males, n=185	Females, n=188	
	Median	Median	
	(Q1–Q3)	(Q1–Q3)	
Variables	(min–max)	(min–max)	p-value
Age (years)	6.5	6.6	0.789
	(6.30–6.80)	(6.30–6.80)	
	(5.9–7.4)	(5.4–7.4)	
Weight (kg)	24.1	23.6	**0.026**
	(22.2–26.7)	(21.50–25.60)	
	(13.6–37.9)	(16.3–42.2)	
Height (m)	1.2	1.2	**<0.001**
	(1.2–1.3)	(1.1–1.3)	
	(1.1–1.4)	(1.2–1.2)	
Body mass index (kg/m^2^)	16.1	16.2	0.990
	(15.3–17.0)	(15.2–17.1)	
	(10.8–23.8)	(12.9–25.0)	
Weight class proportion	%	%	
Thin	2.7(5/185)	4.8 (9/188)	0.289
Normal weight	84.9 (157/185)	76.6 (144/188)	**0.043**
Overweight	9.2 (17/185)	11.7(22/188)	0.430
Obese	3.2(6/185)	6.9(13/188)	0.107
Overweight or obese	12.4(23/185)	18.6(35/188)	0.186

**Table 2.** The prevalence of dental caries (dft > 0) and body mass index-for-age weight classes.

Weight class	Thin	Normal	Overweight	Obese	p-value*	Total
Dental caries (%)	50.0 (7/14)	55.5 (167/301)	64.1 (25/39)	73.7 (14/19)	0.063	57.1 (213/373)

*χ^2^ trend test.

## Discussion

More than half of the children in this study was found to have at least one tooth affected by caries, which underlines the fact that oral health remains a serious health issue in Greenland. Yet the prevalence of caries in this study of 57.1% is much lower than was reported 10 years earlier. In 2005, Petersen and Christensen found the caries prevalence rate among Greenlandic children to be as high as 80–90% [27], which is comparable to the Canadian Inuit community, where over 85% of pre-schoolers in 2008–2009 were found to have caries [28]. A recent study from 2015 found positive effects on caries prevalence among 3- and 9-year-old children after implementation of the National Caries Strategy in Greenland in 2007 [9]. In addition, a national study of 6-year-olds in 2015 is underway and will hopefully show further evidence of improving oral health among Greenlandic children [9]. The percentage of caries-free 5-year-olds in Denmark has risen from as low as 61% in 1993 to 87% in 2014, and only 3% of these 7-year-olds had caries in their permanent teeth in 2014 [29]. This inspiringly high number of caries-free children shows that there is hope for even greater eradication of caries among Greenlandic children.

The prevalence of overweight in this study (11.4% for boys and 19.3% for girls) corresponds well to findings of other recent studies of Greenlandic children. Rex et al. [4] found 19.1% of the girls and 12.7% of the boys in Greenland born in 2005 to be overweight, and Niclasen et al. found that 16.6% of the children in Nuuk born in the years 1997–2001 were overweight [30]. Our study confirms these findings of an increasing problem with overweight among Greenlandic children.

As the figures in Table 2 show, in this study there was a clear tendency for a higher prevalence of caries in overweight and obese children. With a p-value of 0.06, this trend narrowly misses conclusive significance. This tendency might be confirmed in a larger study of Greenlandic children, but there is also a likelihood that the association between overweight and caries is not as evident as their common risk factors would suggest. A large review including 47 studies published from 2004 to 2011 found no significant relationship between weight categories and caries in almost half of the studies (48%) [17]. In another review, significant correlations between caries in permanent teeth and overweight/obesity and between caries and underweight were reported, suggesting a non-linear relationship between weight and dental status [16]. As described above, several factors predisposing to both overweight and caries are lifestyle related and are known causes of both conditions. However, the geographically and genetically determined basis that gives some individuals a vulnerability to develop one or both of the diseases while their peers do not might vary substantially between populations. Among children in Nuuk, Greenland, the results of this study indicate that there is a correlation between overweight and caries that is strong enough to consider and motivate further investigation in this field.

The major strength of this study is that it is the first study to describe the possible association between weight class and prevalence of dental caries in Greenland. However, several limitations exist, and the results must be taken with some reservations. First of all, only 55% of the children in Nuuk were included, since information about either weight, height or prevalence of dental caries was missing. The excluded children did not differ in gender composition compared to the included group, but it cannot be ruled out that children who did not participate in the routine health care checks might have been different from the included children, leading to a possible selection bias. A previous analysis of children without registration of height and weight at school enrolment in Nuuk reported that more than 50% of the missing cases could be explained by migration [4]. This could indicate that the reason for missing data is mainly due to relocation rather than, for example, reluctance to participate, miscommunication or parental neglect, reducing the significance of a possible selection bias. However, as a result of the limited number of children included, the statistical power was consequently reduced. Secondly, this study only included children in Nuuk. Greenland is the world’s largest island, and with extreme geographic distances come differences in culture and lifestyle that might be reflected in health-related issues. Rex et al. [4] found that boys in Nuuk have a slightly lower median BMI compared to their peers in the rest of the country, but found no significant difference in overweight among children in Nuuk compared to other parts of Greenland. Thirdly, at the time of data extraction, we were only able to study the weight, height and dental status at a single point in time for the included children. Among children in Greenland, we do not yet know if childhood overweight and obesity predispose to caries in adolescence or adult life, or if children in Greenland with caries have a tendency to become overweight later in life. With the ongoing implementation of national EMRs in Greenland, data will soon be available in order to analyse possible correlations in this area. In further studies in this field, it would be optimal to screen the study population for comorbidities or special needs, such as diabetes or Crohn’s disease. Such conditions might alter oral health and have an impact on the weight class, thereby distorting the results of the study if data from affected and unaffected individuals are pooled. Since such conditions are very rare among children in the age groups included in this study, the possible effect on the results is not considered to have significantly impacted the conclusions.

In conclusion, we observed an increasing prevalence of caries with increasing weight class among children in Nuuk, Greenland. Yet a linear trend could not be confirmed statistically. The observed high prevalence rates of caries in particular, but also of overweight, indicate a need for a continued focus on preventive initiatives and monitoring. A combined, intensified strategy targeting both caries and overweight may be considered.
